# Insights into the Complexity and Functionality of Hepatitis C Virus NS5A Phosphorylation

**DOI:** 10.1128/JVI.03017-13

**Published:** 2014-02

**Authors:** Douglas Ross-Thriepland, Mark Harris

**Affiliations:** School of Molecular and Cellular Biology, Faculty of Biological Sciences and Astbury Centre for Structural Molecular Biology, University of Leeds, Leeds, United Kingdom

## Abstract

The hepatitis C virus nonstructural NS5A protein has roles in genome replication, virus assembly, and modulation of host pathways. NS5A is a phosphoprotein, and it has been proposed that differential phosphorylation could regulate the various roles of the protein. By SDS-PAGE, two forms of NS5A with distinct apparent molecular weights can be observed, referred to as basally phosphorylated and hyperphosphorylated species. However, the sites of phosphorylation on these two species have not been unequivocally identified, hampering our understanding of the function and regulation of NS5A. To address this, we purified tagged NS5A from cells harboring a replicating subgenomic replicon and analyzed the purified protein by mass spectrometry. We identified six peptide fragments containing 12 phosphorylated residues and were able to assign four of these to serines 146, 222, and 225 and threonine 348. A serine-rich peptide fragment spanning residues 221 to 240 was highly phosphorylated. Using mutagenesis, we identified roles for a subset of these phosphoacceptors in virus genome replication. We further showed that phosphorylation at S146 regulates hyperphosphorylation, and by generating a phospho-specific antibody targeted to pS222, we identified that S222 phosphorylation predominates in the hyperphosphorylated species. Last, by introducing phosphomimetic mutations across residues 221 to 240, we demonstrated changes in the mobility of the basally phosphorylated species suggestive of a sequential phosphorylation cascade within this serine-rich cluster. We propose that this regulation could drive a conformational switch between the dimeric structures of NS5A and could also explain the different functions of the protein in the virus life cycle.

## INTRODUCTION

Hepatitis C virus (HCV) has infected an estimated 3% of the world's population (170 million individuals); in 85% of cases, the virus establishes a chronic infection leading to liver cirrhosis and hepatocellular carcinoma (1). HCV has a 9.6-kb positive-sense, single-stranded RNA genome coding for an ∼3,000-amino-acid polyprotein that is cleaved co- and posttranslationally by both host and viral proteases to produce 10 mature viral proteins. At the N terminus of the polyprotein are the structural proteins, Core, E1, and E2, which make up the virus particle. These are followed by the viroporin p7, which has roles in virus assembly and release (2), and the nonstructural protein 2 (NS2), which contains an autoprotease activity that cleaves it from NS3 but also has a poorly defined role in virion morphogenesis (3, 4). The remaining NS proteins, NS3, NS4A, NS4B, NS5A, and NS5B, are responsible for the replication of the viral genome, but more recently, with the advent of a full-length clone of a genotype 2a isolate (JFH-1) able to undergo the complete virus life cycle in cell culture, these proteins have been shown to play roles in virion assembly and release.

**FIG 1:**
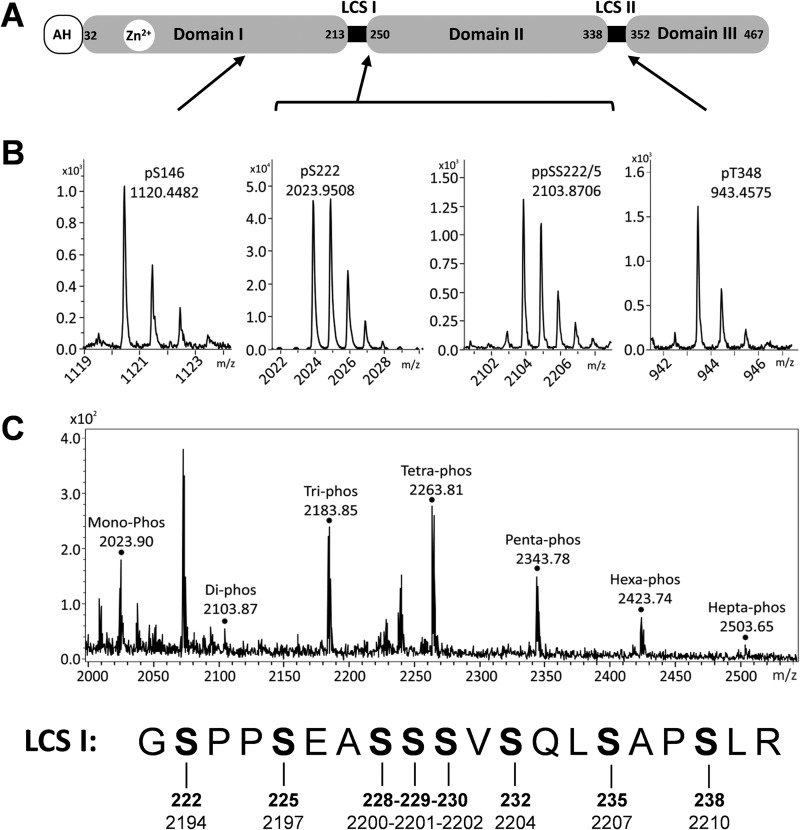
Mass spectrometric analysis of NS5A phosphorylation. (A) Schematic of NS5A structure showing amino acid residue numbers for JFH-1. AH, amphipathic helix; LCS: low-complexity sequence. (B) Mass spectra of NS5A phosphopeptides after analysis by high-performance liquid chromatography (HPLC)-MS/MS. The main monoisotopic peaks were selected for collision-induced dissociation (MS/MS) to allow the precise assignment of peptide and the phosphorylated residues (see Fig. S1 to S4 in the supplemental material). The locations of the four phosphopeptide species within NS5A are indicated. (C) The mass spectra of the NS5A phosphopeptides corresponding to the highly phosphorylated LCS I region, coeluted from the HPLC, resulting in simultaneous observation of all species. Peaks identified as mono- to heptaphosphorylated species are indicated. The sequence of this peptide, together with the amino acid sequence numbering for JFH-1 NS5A (bold) and the JFH-1 polypeptide, is presented below. Monoisotopic peaks for the tri, tetra, and penta species were selected for MS/MS; peptide sequence could be assigned, but exact mapping of phosphorylated residues was hindered by the presence of multiple isomers of each species. Residues S229, S230, S232, and S235, however, were observed as phosphorylated but in unassignable conformations. No MS/MS data could be acquired for the hexa- or heptaphosphorylated species.

In this regard, the NS5A protein has clearly been shown to play key roles in both genome replication and virion assembly/release, but as yet the precise nature of these two functions of the protein and how they are regulated remain to be elucidated. NS5A is comprised of an N-terminal amphipathic helix that anchors the protein to cytoplasmic membranes, followed by three domains linked by two low-complexity sequences (LCS) (see Fig. 1A) that are either serine or proline rich (termed LCS I and II, respectively). Domain I is a highly structured, zinc binding domain whose three-dimensional structure shows two different dimeric conformations (5, 6). Domains II and III have been shown to be natively unstructured, but nuclear magnetic resonance (NMR) and circular dichroism (CD) show that both these domains have elements of secondary structure throughout (7–9). NS5A is a phosphoprotein, and it is widely accepted that NS5A exists as two forms with slightly different mobilities on an SDS-PAGE gel—these have been termed basally phosphorylated (apparent molecular mass of 56 kDa) and hyperphosphorylated (58-kDa) species. The identities of the kinases that phosphorylate NS5A and the sites of phosphorylation remain to be unambiguously determined. A range of kinases have been reported to phosphorylate NS5A either *in vitro* or *in vivo*; these include casein kinases I and II (CKI and CKII) (10–12), polo-like kinase 1 (Plk1) (13), glycogen synthase kinase 3 (GSK3), and mitogen-activated protein kinases (MAPK) (14; for a review, see reference 15). A number of early studies provided evidence for the identities of the phosphorylation sites on NS5A; for example, it was shown (16) that mutation of serines at residue 2197, 2201, or 2204 (polyprotein numbering, corresponding to 225, 229, and 232 in NS5A) resulted in a loss of the hyperphosphorylated species in Cos7 cells transfected with a plasmid expressing NS2-NS5B. These residues are located within LCS I and are absolutely conserved throughout all isolates of HCV. Two studies used mass spectrometry analysis of either glutathione *S*-transferase (GST)-tagged NS5A (GST-NS5A) expressed using a vaccinia-T7 system in BHK21 cells (17) or purified baculovirus-expressed NS5A phosphorylated *in vitro* (18) to identify putative phosphorylation sites. Both of these studies used a genotype 1b NS5A isolate and identified single phosphorylation sites at S2321 (349 in NS5A) or S2194 (222 in NS5A), respectively. It is important to note that these studies involved expression of NS5A in the absence of other viral proteins. Given the fact that various studies have shown that hyperphosphorylation is dependent on other nonstructural proteins (19–21), clearly these data should be interpreted with caution.

Since these early reports, the phenotypes of mutations in these putative phosphorylation sites have been investigated in the context of both subgenomic replicons and full-length JFH-1 virus. These data are difficult to interpret, but a consensus has emerged that in the context of genotype 1b subgenomic replicons, hyperphosphorylation negatively regulates RNA replication (22, 23). However, although the same mutations also abolish hyperphosphorylation in genotype 2a, the negative regulation of RNA replication was not observed (24).

The Tellinghuisen group has shown, using mutagenesis and pharmacological inhibitors, that a serine at residue 457 is a potential CKII phosphorylation site that may play a role in virus assembly (12), although they did not provide biochemical (mass spectrometric) evidence for this. More recently, they demonstrated by mass spectrometry that S222 was a major phosphorylation site in a JFH-1 subgenomic replicon and that mutation of this residue to aspartic acid (as a phosphomimetic) had a modest inhibitory effect on RNA replication (25). A phosphoablatant mutation at S222 (to alanine) also had a modest inhibitory effect on hyperphosphorylation.

Taken together, these data point to the absolutely conserved serine cluster in LCS I as the major site for hyperphosphorylation in NS5A; however, given that NS5A is both serine and threonine rich (JFH-1 NS5A contains 48 serines and 37 threonines: nearly 20% of the protein), there is a great propensity for additional phosphorylation events elsewhere on the protein. Phosphorylation has been proposed to act as a molecular switch regulating the different functions of NS5A (22), although there is as yet no direct experimental evidence to support this hypothesis.

A comprehensive understanding of the phosphorylation and function of NS5A will require the identification of physiologically relevant phosphorylation sites, i.e., those present on NS5A in cells actively replicating viral RNA. To address this issue, we have therefore undertaken a mass spectrometric analysis of JFH-1 NS5A purified on a large scale from Huh7 cells harboring an NS3-5B subgenomic replicon. As expected, this analysis revealed multiple phosphorylation sites, and we have complemented this analysis with mutagenesis to determine the phenotypes of these phosphorylation events in the context of both subgenomic replicons and virus. Our data reveal a complex pattern of phosphorylation within LCS I and provide evidence for a sequential cascade of phosphorylation across this region and for regulation by a distal phosphorylation event within domain I.

## MATERIALS AND METHODS

### DNA constructs.

DNA constructs of either the full-length pJFH-1 virus (26), luciferase reporter subgenomic replicon (SGR-luc-JFH-1) (27), or neomycin reporter SGR with NS5A containing a One-Strep tag (1ST) (pSGR-5A1ST) (28) were used throughout. Previously, unique restriction sites flanking NS5A, BamHI and AfeI, were introduced into both full-length virus and SGR-luc-JFH-1 constructs; these constructs were denoted as mJFH-1 and mSGR-luc-JFH-1, respectively, and shown to have no effect on virus genome replication or assembly and release (29). Mutagenesis of NS5A was performed on a LITMUS28i (NEB) subclone containing an NsiI/HindIII fragment of the mJFH-1 cDNA by QuikChange site-directed mutagenesis (Stratagene) and then cloned into either mSGR-luc-JFH-1 or mJFH-1 via the flanking BamHI/AfeI sites. All mutations were verified by sequencing; plasmid and primer sequences are available on request.

### Cell culture.

Huh7 cells were cultured in Dulbecco's modified Eagle's Medium (DMEM) (Sigma) supplemented with 10% fetal bovine serum (FBS), 100 IU penicillin/ml, 100 μg streptomycin/ml, and 1% nonessential amino acids in a humidified incubator at 37°C, 5% CO_2_. For virus propagation, medium was supplemented with 25 mM HEPES. Replicon-harboring cells containing a neomycin resistance gene were selected for 20 days postelectroporation at 1 mg/ml G418 (Sigma) before passaging at 1:5 every 3 days under 0.5-mg/ml G418 selection.

### Purification of NS5A.

A stable Huh7 cell line harboring the SGR-5A1ST subgenomic replicon was passaged as described above. At each passage, excess cells were washed twice in phosphate-buffered saline (PBS), pelleting at 500 × *g*, 5 min at room temperature, before lysis in Glasgow lysis buffer (30) (GLB) [1% Triton X-100, 120 mM KCl, 30 mM NaCl, 5 mM MgCl_2_, 10% glycerol, and 10 mM piperazine-*N*,*N*′-bis(2-ethanesulfonic acid) (PIPES)-NaOH, pH 7.2] containing protease and phosphatase inhibitors, 2 ml per 175-cm^2^ flask. Lysates were clarified by centrifugation at 4,000 × *g*, 5 min at 4°C, and filtered through a 0.45-μm cellulose filter before batch affinity purification of NS5A-1ST on Strep-Tactin Sepharose columns (IBA), with a 5-ml column volume, following the manufacturer's instructions. Bound protein was washed with 1 column volume of wash buffer supplemented with low salt (0.5 M NaCl) or high salt (1 M NaCl and 1% Triton X-100) for three and four washes, respectively, before two standard washes and competitive elution with 2.5 mM desthiobiotin at 0.5 column volume. Peak elution fractions, 3 and 4, were identified by Western blotting and concentrated, first by centrifugation filtration (10,000 [10K]-molecular-weight cutoff) followed by methanol-chloroform precipitation. The final precipitate was resuspended in 20 μl of 1× SDS-LB and separated further on a 12% Tris-glycine SDS-PAGE gel. After staining with Coomassie R250, the band corresponding to NS5A was excised for mass spectrometry analysis.

### Mass spectrometry analysis.

An excised gel slice was sectioned in two and subjected to in-gel digestion with either GluC or trypsin, following standard protocols. Peptide fragments eluted from gel slices and phosphopeptides were enriched by titanium dioxide enrichment. Samples were analyzed by liquid chromatography-electrospray ionization-tandem mass spectrometry (LC-ESI-MS/MS) or LC–matrix-assisted laser desorption ionization–MS/MS (LC-MALDI-MS/MS) as denoted, and resulting MS and MS/MS spectra were interrogated by using the software program Mascot (Matrix Science), with identified phosphopeptides confirmed manually.

### *In vitro* transcription.

DNA constructs were linearized by XbaI, mung bean nuclease treated, and phenol-chloroform extracted. Linearized DNA was then used as the template in a RiboMAX reaction (Promega). RNA transcripts were purified by phenol-chloroform extraction, quantified by absorbance at 260 nm, and analyzed by agarose gel electrophoresis to confirm integrity and quantification.

### Luciferase-based replication assay.

Huh7 cells were washed twice in diethylpyrocarbonate (DEPC)-treated PBS before electroporating 4 × 10^6^ cells in DEPC-PBS with 2 μg of RNA at 950 μF and 270 V. Cells were resuspended in complete medium before being seeded into both 96-well plates (*n* = 6) at 3 × 10^4^ cells/well and 6-well plates (*n* = 2) at 3 × 10^5^ cells/well, both plates incubated under cell culture conditions. At 4 and 72 h postelectroporation (hpe), cells were harvested by lysis with either 30 μl or 200 μl passive lysis buffer (PLB) (Promega), 96- and 6-well, respectively. Luciferase activity was determined from 96-well samples on a BMG plate.

### Virus replication and release assay.

Huh7 cells were washed twice in DEPC-PBS before electroporating 2 × 10^6^ cells in DEPC-PBS with 1 μg of RNA at 960 μF and 270 V. Cells were resuspended in complete medium and seeded at 10^6^ cells/well into 6-well plates. At 72 hpe, cells were split at 1:5 before incubating for a further 72 h. At 144 hpe, cells were harvested in 400 μl TRIzol reagent for real-time quantitative PCR (qRT-PCR) analysis, while supernatants were removed and clarified at 2,800 × *g* for 5 min at room temperature before storing at −80°C.

### Virus titer determination by focus-forming assay.

Titers of clarified virus supernatants were determined on Huh7 cells as follows. In a 96-well format, clarified virus supernatants were serially diluted 5-fold in complete DMEM medium plus HEPES before the addition of 100 μl diluted virus to Huh7 cells seeded 24 h previously into 96-well plates at 8 × 10^3^ cells/well, with a final volume of 200 μl. Cells were incubated under normal cell culture conditions for 72 h before fixation, staining, and focus counting as described previously (31).

### RNA extraction and qRT-PCR.

To quantify the number of HCV genomes, total cell RNA was extracted by TRIzol, following the manufacturer's instructions (Invitrogen). Total extracted cellular RNA, 100 ng, was analyzed by qRT-PCR using a one-step qRT-PCR TaqMan-based kit as directed (Eurogentec), with primers and probe designed against the 5′ untranslated region (UTR) as described previously (32).

### SDS-PAGE/Western blotting.

Cells were washed twice with PBS, lysed by resuspension in GLB containing protease and phosphatase inhibitors, and incubated on ice for 15 min. Insoluble material was pelleted by centrifugation at 500 × *g* for 5 min at 4°C. Following separation by SDS-PAGE, proteins were transferred to a polyvinylidene difluoride (PVDF) membrane and blocked in 50% Odyssey blocking buffer (Li-Cor) in PBS. The membrane was incubated with primary antibody in 50% Odyssey blocking buffer and then incubated with fluorescently labeled anti-goat (800 nm) or anti-mouse (700 nm) before imaging on a Li-Cor Odyssey Sa infrared imaging system. Primary antibodies used were as follows: NS5A (9E10), 1:5,000 (a kind gift from Tim Tellinghuisen); NS5A (sheep), 1:10,000 (30); Core (sheep), 1:5,000 (in house); glyceraldehyde-3-phosphate dehydrogenase (GAPDH), 1:30,000 (Sigma).

### Quantification of basal/hyperphosphorylation.

Quantification of fluorescently labeled Western blots was carried out using Image Studio v3.1 software (Li-Cor) using a background subtraction method.

### Validation of phospho-specific pS222 antibody.

For Western blotting, PVDF membranes were blocked in 5% (wt/vol) bovine serum albumin (BSA)-Tris-buffered saline (TBS) for 1 h at room temperature before incubation with primary antibody at 1 μg/μl with 10 μg/ml nonphosphorylated 13-mer peptide in 50% Odyssey blocking buffer in TBS. Note that antibody/nonphosphorylated peptide was preincubated for 1 h at room temperature.

### Statistics.

Data sets analyzed using Student's *t* test assuming a two-tailed, unequal variance to determine the statistical difference from results for the wild type (WT) (*n* = 3 or greater throughout; *, *P* < 0.05; **, *P* < 0.01).

## RESULTS

### Identification of NS5A phosphorylation sites by mass spectrometry.

NS5A is known to be highly phosphorylated; however, with the exception of a recent study (25), the site(s) of phosphorylation on NS5A expressed in a physiologically relevant context, i.e., in human hepatocytes either infected with HCV or harboring subgenomic replicons, has not been definitively determined. To address this gap in our understanding, we took advantage of the fact that domain III of NS5A is able to tolerate in-frame insertions with no effect on the functions of the protein in virus RNA replication or assembly. Specifically, we utilized a subgenomic replicon in which NS5A was tagged with the One-Strep affinity tag (1ST); the insertion of this short tag had been previously shown not to affect RNA replication (28) and provided a highly efficient and selective purification strategy. NS5A-1ST was affinity purified using Strep tag beads from cytoplasmic lysates of approximately 5 × 10^9^ cells prior to separation by SDS-PAGE. After staining of the gel with Coomassie brilliant blue R250, the band corresponding to NS5A was excised and subjected to either trypsin or GluC cleavage, TiO_2_ enrichment of phosphorylated peptides, and mass spectrometry analysis by MS-MS utilizing both MALDI and ESI approaches to identify phosphorylated species.

This analysis allowed the unambiguous assignment of four phosphorylation sites on different phosphopeptides (Fig. 1A and B and Table 1; see also Fig. S1 to S4 in the supplemental material): first, a peptide located within domain I in which serine 146 was phosphorylated; second, two peptides located within the serine-rich LCS I between domains I and II (one of these peptides contained a single phosphoserine at residue 222, and the other contained two phosphoserines at residues 222 and 225); the fourth peptide was located in the proline-rich LCS II between domains II and III and contained a phosphothreonine at residue 348.

**TABLE 1:** NS5A phosphopeptides identified by mass spectrometry

Peptide fragment observed by mass spectrometry^*a*^	No. of phosphorylation events; location(s)^*b*^	[M+H]^+^		Type of MS analysis used		
		Calculated	Observed	MALDI	ESI	MS/MS
KAP**pT**PPPR	1; T348	943.4761	943.4575	Yes	No	Yes
IPCQLP**pS**PE	1; S146	1120.4743	1120.4482	Yes	Yes	Yes
G**pS**PPSEASSSVSQLSAPSLR	1; S222	2023.9332	2023.9508	Yes	Yes	Yes
G**pS**PP**pS**EASSSVSQLSAPSLR	2; SS222/225	2103.8995	2103.8706	Yes	Yes	Yes
GSPPSEASSSVSQLSAPSLR	3; multiple isomers	2183.8659	2183.8477	Yes	No	Yes
GSPPSEASSSVSQLSAPSLR	4; multiple isomers	2263.8322	2263.811	Yes	No	Yes
GSPPSEASSSVSQLSAPSLR	5; multiple isomers	2343.7985	2343.7756	Yes	No	Yes
GSPPSEASSSVSQLSAPSLR	6; unassigned	2423.7648	2423.7419	Yes	No	No
GSPPSEASSSVSQLSAPSLR	7; unassigned	2503.7311	2503.7082	Yes	No	No
ATCTTHSNTYDVDMVDANLLMEGGVAQTEPESR	1; T242 or T244 or T245 or S247 or T249	3724.5449	3724.5441	No	Yes	Yes
TFGQPPSSGDAGSSTGAGAAE	1; S387 or S388	1931.7655	1931.7629	No	Yes	Yes
SGGPTSPGEPAPSE	1; S396 or T400 or S401	1349.5257	1349.5414	Yes	No	Yes

a Bold and underlining in sequences indicate phosphorylated residues and putative phosphorylated residues respectively.

b JFH-1 NS5A protein numbering.

A number of other phosphopeptides mapping to the LCS I were identified by MS/MS to be extensively phosphorylated, containing between 3 and 7 phosphorylated residues (Fig. 1C). The exact arrangement of phosphorylations in these peptide species could not be identified. In the case of the tri-, tetra-, and pentaphosphorylated species, MS/MS data showed that S229, S230, S232, and S235 were phosphorylated residues but that these phosphorylation events were occurring in an arrangement of different, unassignable isomers. In the case of the hexa- and heptaphosphorylated species, the ion suppression effects caused by multiple phosphorylations resulted in insufficient ion abundance for MS/MS data to be collected; as such, no assignment could be carried out. For similar reasons, the presence of an octaphosphorylated species of the peptide fragment spanning 221 to 240, cannot be excluded. Our data point to a complex pattern of phosphorylation in this region of NS5A, and this concept is explored further later in the article.

Three other phosphopeptides were also identified as containing a single phosphorylated residue—these corresponded to peptides 241 to 273, 375 to 395, and 396 to 409 (Table 1). The MS/MS data were not sufficient to allow the unambiguous assignment of the phosphorylated reside in each phosphopeptide, but was able to identify a subset of serines/threonines at which phosphorylation was occurring. Due to the uncertainties resulting from the analysis of these phosphopeptides, this study instead focused on those phosphoacceptors for which evidence was more conclusive.

### Role of NS5A phosphorylation in HCV life cycle.

**FIG 2:**
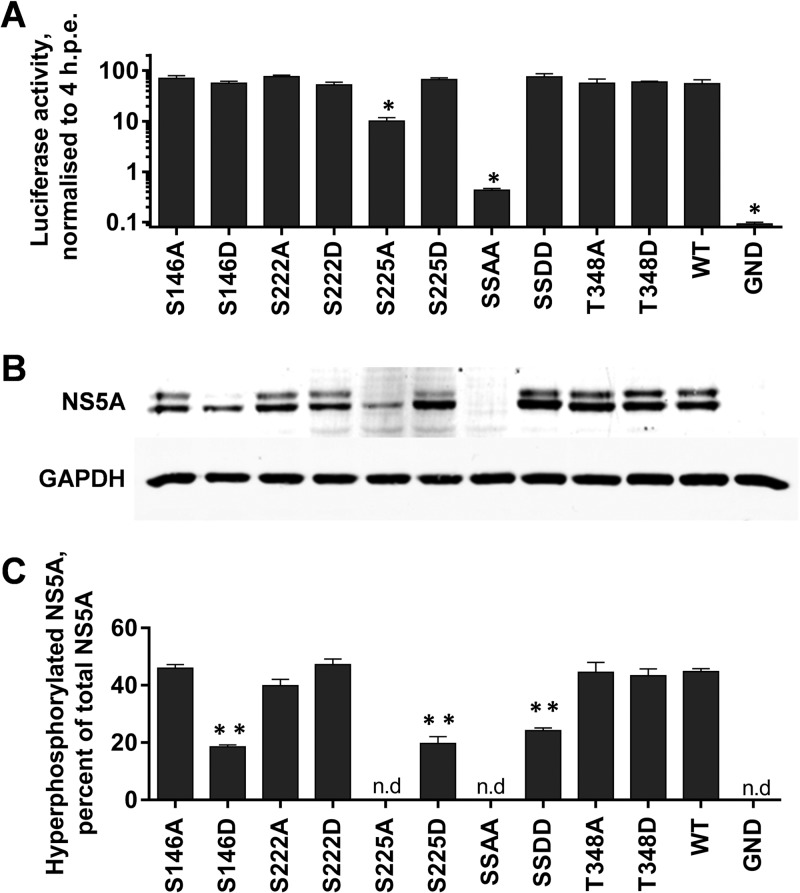
Mutagenic analysis of phosphorylation in the context of the HCV subgenomic replicon. The indicated mutations were engineered into mSGR-Luc-JFH-1 (29), *in vitro* transcribed RNA was electroporated into Huh7 cells, and luciferase activity was measured at 4 and 72 hpe. A polymerase-inactive mutant (GND) was included as a negative control. (A) Luciferase activity at 72 hpe is shown (*n* = 3) normalized to activity at 4 h. *, *P* < 0.05, significance difference from the wild type. (B) Western blot analysis of cell lysates at 72 hpe probed with NS5A (9E10) or GAPDH antibodies. Use of a 7.5% SDS-PAGE gel allowed the separation of the basally (lower band) and hyperphosphorylated (upper band) species of NS5A. (C) Quantification of the percentage of total NS5A that is hyperphosphorylated from Western blot. Western blots were imaged using a Li-Cor Odyssey Sa infrared imaging system, enabling highly accurate quantification (*n* = 3). **, *P* < 0.01, significant difference from the wild type. n.d, no data due to low levels of expression. “SSAA” refers to the S222/225A double mutant; “SSDD” refers to the corresponding S222/225D mutant.

To investigate the role of the four unambiguously identified phosphorylation sites in the virus life cycle, each residue was mutated to either alanine to block phosphorylation (phosphoablatant) or aspartic acid to introduce a negative charge, thereby acting as a phosphomimetic. These mutations were introduced into both a luciferase-based subgenomic replicon (SGR-luc-JFH-1) or a full-length JFH-1 virus construct, allowing us to assess the role of phosphorylation at different stages of the virus life cycle. In addition to the four single site mutations, the two serines at 222 and 225 were mutated simultaneously. Figure 2A shows the results of this mutagenesis in the context of the subgenomic replicon. Neither the phosphoablatant nor phosphomimetic mutations at serines 146 and 222 (S146A/D and S222A/D) or threonine 348 (T348A/D) had any significant effect on RNA replication. All of the resulting mutated subgenomic replicons were able to replicate with equal efficiency (∼100-fold increase in luciferase expression over 72 h postelectroporation [hpe]). In contrast, the S225A phosphoablatant mutation resulted in a 10-fold reduction in replication, whereas an S225D mutant exhibited wild-type levels of replication, suggesting that phosphorylation of this residue is important for HCV genome replication. Consistent with this, the double alanine substitution (S222/225A) almost completely abolished the replicative ability of the subgenomic replicon, with levels of luciferase activity at 72 hpe only slightly higher than those of the GND negative control (which contains an inactivating mutation in the NS5B polymerase). The corresponding double aspartic acid substitution (S222/225D) had wild-type activity. We conclude that S222 phosphorylation makes a modest contribution to genome replication which is apparent only in the context of a further phosphorylation event at S225.

By Western blotting, wild-type NS5A appears as a doublet of what have been referred to as basally and hyperphosphorylated species. Of note, although these two species are commonly referred to as p56 and p58, in JFH-1, since there is an 18-amino-acid insertion in domain III compared to genotype 1b (29), they migrate with apparent molecular masses of 63 and 65kDa (see Fig. S5A in the supplemental material). For clarity in this article, we will refer to them as the basally and hyperphosphorylated species of NS5A. To assess the potential effects of these mutations on NS5A expression and the relative abundances of these two species, lysates from cells electroporated with subgenomic replicon RNAs were analyzed by Western blotting (Fig. 2B). In most cases, there was no difference in the expression profile of NS5A compared to that for the wild type. Where possible, the percentage of hyperphosphorylated NS5A was determined (Fig. 2C), although the low levels of NS5A expression in the poorly replicating S225A and S222/225A mutants did not allow quantitation. Surprisingly, the S225D and S222/225D mutants exhibited a reduction in hyperphosphorylation, although these residues (along with S229 and S232) have previously been proposed as phosphoacceptor sites in the hyperphosphorylated species of NS5A. This suggests that an aspartate residue may mimic the charge but not the structural effects of phosphorylation. Intriguingly, whereas the S146D mutant exhibited a reduction in hyperphosphorylation, the S146A mutant exhibited wild-type levels of hyperphosphorylated NS5A, suggesting that while phosphorylated S146 is not a component of hyperphosphorylated NS5A, it does negatively regulate hyperphosphorylation.

**FIG 3:**
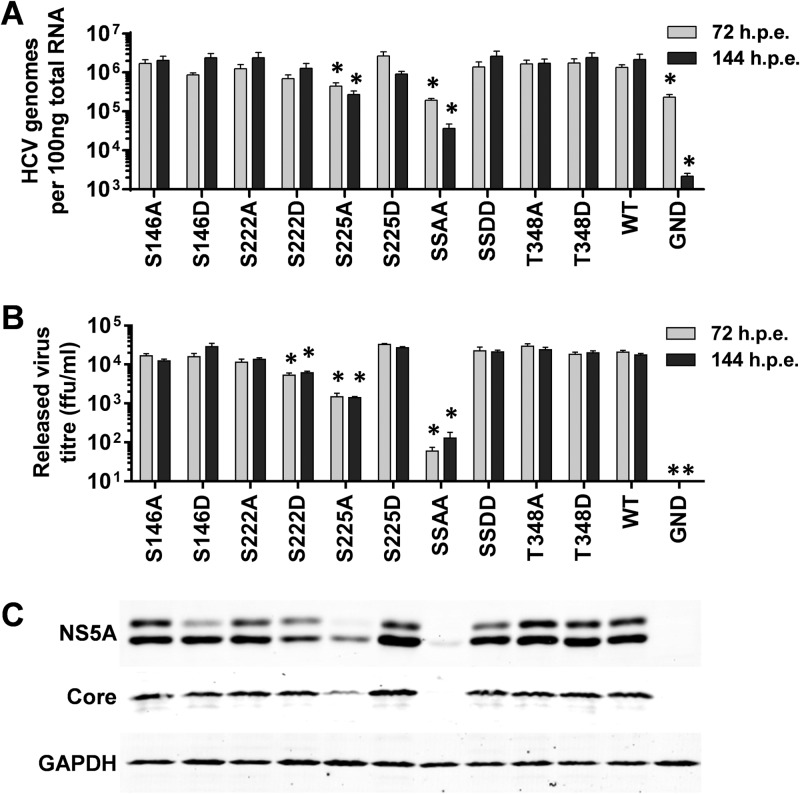
Mutagenic analysis of phosphorylation in the context of JFH-1 virus. The indicated mutations were engineered into mJFH-1 (29), and *in vitro*-transcribed RNA was electroporated into Huh7 cells. Cells were cultured for 144 h, including a 1:5 passage at 72 hpe, total RNA was extracted from cells at 72 and 144 hpe, and HCV genomes were quantified by qRT-PCR using primers targeted to the 5′ UTR (31) (*n* = 3). *, *P* < 0.05, significant different from the wild type. HCV genomes for the nonreplicating control, GND, were still detectable, most likely due to the stability of the 5′ UTR. (B) Supernatants were assayed for released infectious virus titer by focus-forming assay at 72 and 144 hpe. ffu, focus-forming units. (C) Lysates were prepared from cells at 144 hpe and probed for NS5A (9E10), Core, and GAPDH. “SSAA” refers to the S222/225A double mutant; “SSDD” refers to the corresponding S222/225D mutant.

A similar set of data was obtained when this series of mutations was evaluated in the context of the full-length JFH-1 infectious clone (Fig. 3). The majority of the mutants showed wild-type levels of both intracellular genome abundance measured by qRT-PCR (Fig. 3A) and titers of released virus measured by focus-forming assay (Fig. 3B). Again the S225A mutant exhibited an approximately 10-fold reduction in both assays, and the S222/225A mutant was almost completely deficient in both virus genome replication and assembly. Intriguingly, the S222D mutant exhibited a modest but statistically significant reduction in released infectivity (Fig. 3B), but this was not seen at the level of genome replication (Fig. 3A). It is therefore possible that S222 phosphorylation could regulate assembly and release of infectious virus by inhibiting the (as yet undefined) role of NS5A in these processes. The Western blot analysis (Fig. 3C) also paralleled that observed for the subgenomic replicons. Although in this case a low level of hyperphosphorylation could be seen in the context of the S146D mutant, this was clearly reduced compared to those of both the wild type and the S146A mutant. We conclude that the phenotype of these mutants is entirely manifest at the level of virus genome replication and there are no additional effects on virion assembly or release.

### Further analysis of phosphorylation within LCS I.

**FIG 4:**
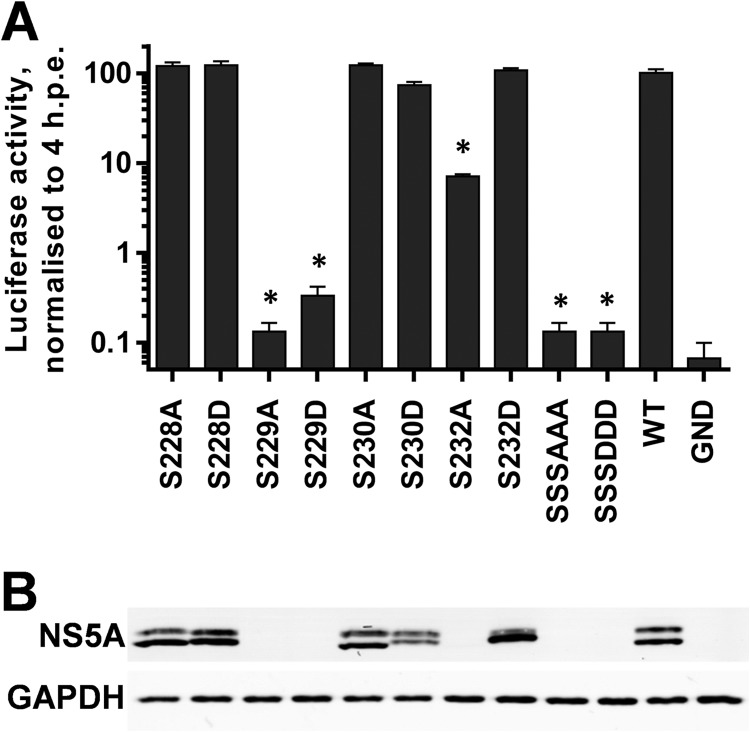
Mutagenic analysis of putative phosphorylation sites within LCS I. The indicated mutations were engineered into mSGR-Luc-JFH-1 (29), *in vitro*-transcribed RNA was electroporated into Huh7 cells, and luciferase activity measured at 4 and 72 hpe. A polymerase-inactive mutant, GND, was included as a negative control. (A) Luciferase activity at 72 hpe is shown (*n* = 3) normalized to activity at 4 h. *, *P* < 0.05, significant difference from the wild type. (B) Western blot analysis of cell lysates at 72 hpe probed with NS5A (9E10) or GAPDH antibodies. “SSSAAA” refers to the S228/229/230A triple mutant, and “SSSDDD” refers to the corresponding S228/229/230D mutant.

In addition to the mono- and diphosphorylated peptides mapping to LCS I (S222 and S222/225), the mass spectrometric analysis revealed evidence for the existence of phosphorylated species containing between three and seven phosphorylation events (Table 1). In order to investigate the potential function of these phosphorylation events, a further set of phosphoablatant and phosphomimetic mutants was generated, and their phenotypes were evaluated in the context of the SGR-luc-JFH-1 subgenomic replicon. As shown in Fig. 4A, S228 and S230 did not appear to have any role in genome replication, since mutations of either of these residues to alanine or aspartic acid had no phenotype. In contrast, S229 was a key residue since both the S229A and S229D mutations abrogated RNA replication. Consistent with this, a subgenomic replicon with a triple mutation of S228/229/230 to either alanine or aspartic acid was also replication defective. S232 resembled S225 in that the S232A mutation reduced replication approximately 10-fold, whereas S232D exhibited wild-type activity. These data point to important roles for S229 and S232 in HCV genome replication; at this stage, it is pertinent to note that these residues were identified as phosphoacceptors in the tri- and tetraphosphorylated peptides but in configurations that could not be unambiguously assigned.

**FIG 5:**
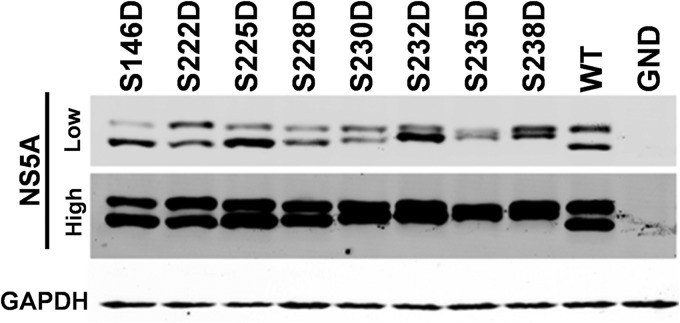
Phosphomimetic mutations in LCS I increase the apparent molecular weight of the basally phosphorylated NS5A species. Lysates from electroporated cells were prepared at 72 hpe and analyzed by Western blotting with antibodies to NS5A (9E10) or GAPDH. Lysates were separated by 7.5% SDS-PAGE at low voltage for an extended time (110 V, 120 min), allowing for optimal resolution of basally and hyperphosphorylated forms. The NS5A Western blot is shown at both low and high exposures.

**FIG 6:**
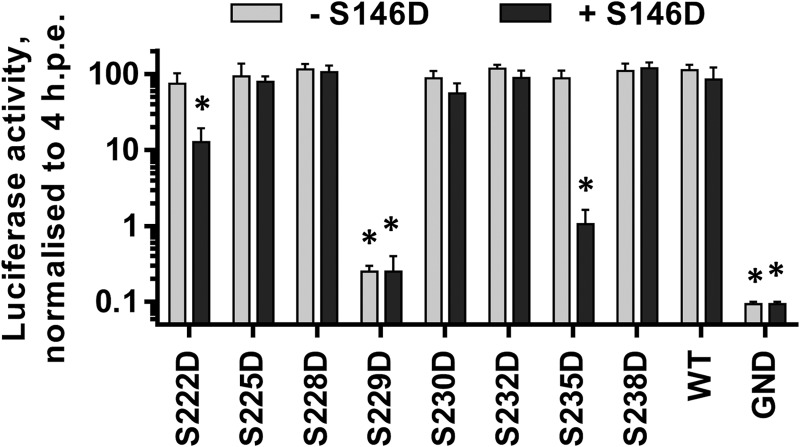
Analysis of LCS I phosphomimetic mutations in the context of S146D. The indicated mutations were engineered into mSGR-Luc-JFH-1 (29), *in vitro*-transcribed RNA was electroporated into Huh7 cells, and luciferase activity measured at 4 and 72 hpe. A polymerase-inactive mutant, GND, was included as a negative control. (A) Luciferase activity at 72 hpe is shown (*n* = 3) normalized to activity at 4 h. For each mutant, the results obtained in either a wild-type (gray bars) or S146D mutant (black bars) background are shown side by side. *, *P* < 0.05, significant difference from the wild type.

Western blot analysis of NS5A expression in cells harboring the subgenomic replicons reflected the levels of replication (Fig. 4B); however, this analysis led to an intriguing observation regarding the mobility of the two phosphorylated forms. Whereas the mobility of the hyperphosphorylated species did not vary, there were differences in the mobilities of the basally phosphorylated species between the various mutants. Specifically, although alanine substitutions did not change the mobility of the basally phosphorylated species compared to that of the wild type, aspartic acid substitutions resulted in a subtle retardation of its mobility. This could also be seen to a lesser extent for mutation S225 and the combination of S222/225 but interestingly not for S146 or T348 (Fig. 2), suggesting that this was not simply due to the substitution of an acidic residue into the protein. To obtain a clearer picture of this phenomenon, we analyzed all of the phosphomimetic mutants capable of replication (therefore excluding the S229D mutant) on a single Western blot (Fig. 5). For completeness, we also included the S235D and S238D mutants, which were also replication competent (Fig. 6). Although the overall levels of expression varied, a clear trend emerged: as the position of the phosphomimetic mutation moved toward the C terminus of this serine cluster, the mobility of the basally phosphorylated species was progressively retarded. One explanation for this result is that phosphorylation of one residue completes the kinase recognition motif for a neighboring residue to be phosphorylated and so on, resulting in a sequential phosphorylation cascade.

### Regulation of hyperphosphorylation by S146.

As shown in Fig. 2 and 3, the S146D mutation resulted in a reduction in the level of hyperphosphorylation, suggesting that phosphorylation of this residue in some way negatively regulated phosphorylation at other sites (possibly within the serine-rich LCS I). We reasoned that if sequential phosphorylation with LCS I contributed to hyperphosphorylation, then the introduction of phosphomimetic mutations might override this effect of S146D. To test this, we generated a series of phosphomimetic mutations in LCS I in the background of the S146D mutation within the SGR-luc-JFH-1 subgenomic replicon and evaluated replication by luciferase assay and NS5A expression by Western blotting. In both assays, we compared the mutant panel in either a wild-type or S146D background.

**FIG 7:**
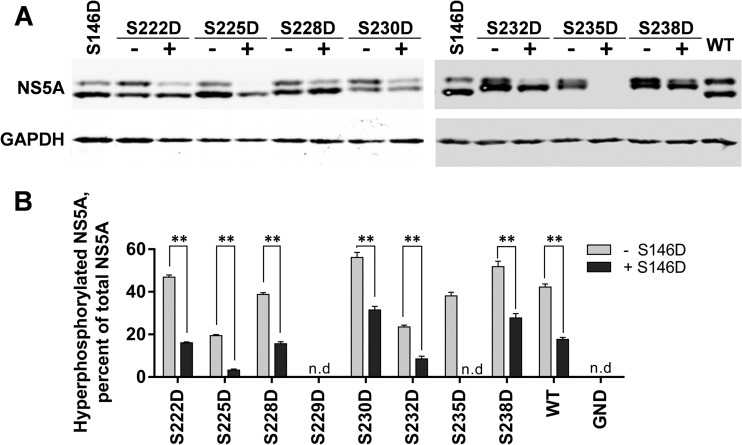
Effect of S146D on LCS I phosphorylation. Lysates from electroporated cells were prepared at 72 hpe and analyzed by Western blotting with antibodies to NS5A (9E10) or GAPDH. Lysates were separated by 7.5% SDS-PAGE at low voltage for an extended time (110 V, 120 min),1 allowing for optimal resolution of basally and hyperphosphorylated species. For each mutant, the results obtained in either a wild-type (-) or S146D mutant (+) background are shown side by side. (B) Quantification of the percentage of total NS5A that is hyperphosphorylated from Western blots. Western blots were imaged using a Li-Cor Odyssey Sa infrared imaging system, enabling highly accurate quantification (*n* = 3). For each mutant, the results obtained in either a wild-type (gray bars) or S146D mutant (black bars) background are shown side by side. **, *P* < 0.01, significant difference from the wild type. n.d, no data due to low levels of expression.

Figure 6 shows that for S225D, S228D, S230D, S232D, and S238D mutants, the addition of the S146D mutation had no effect on replication, since all of these subgenomic replicons were indistinguishable from the wild type. As previously shown (Fig. 4A), the S229D mutant was completely replication defective, and this was not changed by the introduction of the S146D mutation. Two mutations, S222D and S235D, showed an altered phenotype, whereby in the wild-type background these mutations had no effect on replication, but in combination with S146D, viruses carrying both exhibited impaired replicative capacity (approximately 1- or 2-log reductions, respectively). Western blot analysis of cell lysates showed two distinct features. First, the introduction of S146D alongside LCS I mutations (S228D, S230D, S232D, and S238D) did not block their ability to increase the apparent molecular weight of the basally phosphorylated NS5A (Fig. 7A). Second, the presence of any phosphomimetic mutation within LCS I (222–238) did not block the ability of S146D to reduce hyperphosphorylation of NS5A, since in all cases where the double mutants were capable of replication, the presence of S146D reduced the abundance of hyperphosphorylation (Fig. 7B). These data show that the phenotype of both S146 and LCS I phosphomimetic mutations cannot elicit a dominant effect over the other.

### S222 phosphorylation is a hallmark of the hyperphosphorylated NS5A species.

**FIG 8:**
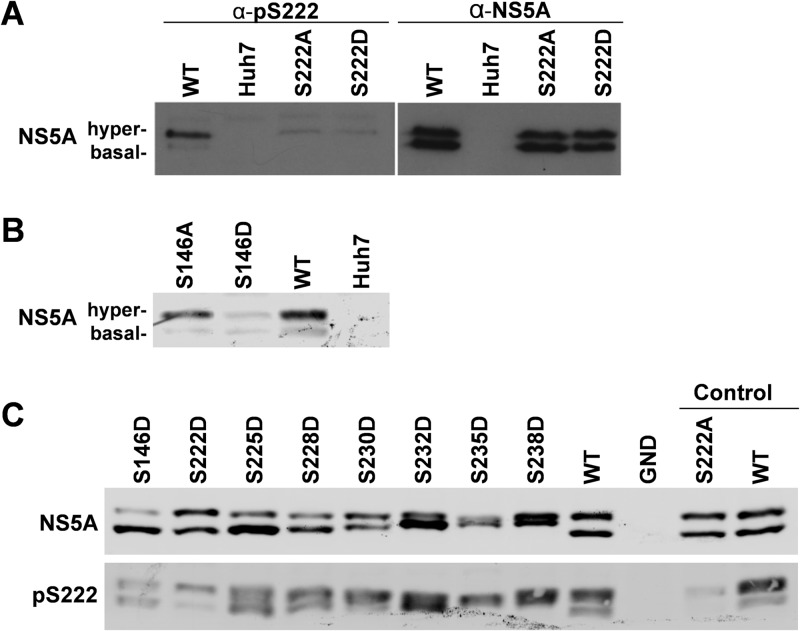
Phosphorylated S222 is predominately in the hyperphosphorylated species. A phospho-specific antibody was raised in sheep against pS222 using an appropriate 13-mer peptide and purified by IgG enrichment. (A) Specificity of the pS222 antibody. Lysates from Huh7 cells electroporated with the indicated subgenomic replicons were analyzed by SDS-PAGE/Western blotting and probed with either anti-pS222 (left panel), or anti-NS5A (right panel) sheep polyclonal antisera. (B) Indicated lysates were probed with the anti-pS222 antiserum. (C) Lysates were probed with anti-NS5A (upper panel) or anti-pS222 (lower panel).

It has been previously suggested that S222 is a critical phosphoacceptor in the formation of the hyperphosphorylated NS5A species. However, the evidence for this is indirect, and differential phosphorylation of S222 between the basally and hyperphosphorylated species was not demonstrated. In order to determine the phosphorylation status of S222 in the two species of NS5A, we raised a sheep polyclonal phospho-specific antiserum using an appropriate phosphopeptide. Figure 8A presents a validation of this reagent and shows that the anti-pS222 antiserum did indeed react only with the hyperphosphorylated NS5A species. A very faint band can be seen corresponding to basally phosphorylated NS5A; however, this is most likely due to weak recognition of the surrounding peptide sequence. Reassuringly, the serum showed only very limited cross-reactivity with the S222A or S222D mutants; again the faint bands are likely due to recognition of the surrounding sequence. This is the first biochemical evidence that S222 phosphorylation is enriched in the hyperphosphorylated NS5A species, although we cannot rule out that it is also present at very low levels in the basally phosphorylated species. Consistent with the loss of the hyperphosphorylated species for the S146D mutant (see Fig. 2B), Western blot analysis using anti-pS222 antiserum revealed that pS222 reactivity was much reduced for this mutant (Fig. 8B). These data provides further confirmation of the role of S146 in regulating phosphorylation within LCS I. Further Western blot analysis also demonstrated that the phosphomimetic S222D to S238D mutants were also able to increase the reactivity of the basally phosphorylated species toward the pS222 antibody (Fig. 8C).

## DISCUSSION

We present in this article a comprehensive analysis of NS5A phosphorylation in a physiologically relevant context, i.e., from Huh7 cells harboring a JFH-1-derived subgenomic replicon. Our study, together with a recent publication (25), represents the only biochemical analyses of NS5A phosphorylation expressed in human cells in the context of the other nonstructural proteins and an active RNA replication complex. Both studies identified S222 within LCS I as a major site of phosphorylation (25). However, whereas Lemay et al. only showed S222 to be a phosphoacceptor, our data identified a large number of other sites of phosphorylation, not only in LCS I but in domains I and III and LCS II. The difference between the two studies is likely due simply to the amount of starting material—LeMay et al. used 7.5 × 10^7^ cells, while our study used approximately 100-fold more, thereby allowing the identification of phosphopeptides that are detected with a lower efficiency by MS (peptides that are large and/or highly phosphorylated), as well as those that are less abundant. To overcome the absence of trypsin cleavage sites in the C-terminal 121 residues of NS5A, we also digested the purified protein with GluC. Despite this, we were still unable to detect the putative phosphoacceptor S457 in domain III (12, 29), which was likely a result of peptides containing that modification being at low abundance or containing multiple phosphorylations. Neither study was able to discriminate between phosphoacceptors present on the basally and/or hyperphosphorylated NS5A species, due to the loss of resolution when separating large amounts of protein by SDS-PAGE. It is important to note that during the propagation of subgenomic replicon-harboring cells for NS5A purification, we endeavored to use cells at a low passage number. The reason for this is that we have routinely observed a loss of the hyperphosphorylated species following long-term passage of replicon cell lines (see Fig. S5 in the supplemental material for an example). Sequence analysis of replicons following long-term passage did not reveal any mutations in NS5A (data not shown), so this is most likely caused by changes in cellular gene expression, although we cannot rule out mutations in other nonstructural proteins.

### Threonine 348: an “orphan” phosphoacceptor with no phenotype.

Our analysis identified T348 within LCS II as a phosphoacceptor. Although T348 is not conserved in other genotypes of HCV (in genotype 1, this residue is a valine or isoleucine), in genotype 1a (strain H), a serine just before the PxxPxR motif was identified as a phosphoacceptor, albeit in the context of expression of a GST-NS5A fusion in BHK21 cells (17). Thus, there is precedent for the existence of a phosphorylatable residue in this region of the protein. Neither a phosphoablatant nor phosphomimetic mutation at this site had any phenotype in replication and/or virus assembly, but it was intriguing that this residue was located in the middle of a well-characterized class II polyproline motif (PxxPxR) that binds to a range of SH3 domains (33, 34). We considered that phosphorylation at T348 might influence the binding of NS5A to SH3 domains; however, as shown in Fig. S6 in the supplemental material, this was not the case. Both the T348A and T348D mutants bound as well as the wild type to a range of GST-SH3 domain fusion proteins in an *in vitro* GST pulldown assay using lysates from subgenomic replicon-harboring Huh7 cells. In contrast, the PA2 mutant (in which the prolines were substituted for alanines) failed to bind to GST-SH3 fusions. Thus, the potential role of this phosphorylation event remains elusive.

### LCS I is the predominant location for phosphorylation of NS5A.

The majority of phosphoacceptor residues identified in this study were in LCS I, between S222 and S238. Remarkably, this serine-rich cluster is absolutely conserved across all genotypes of HCV, implying an essential role in the virus life cycle. A 19-residue tryptic peptide covering this region contained 8 serines, and our mass spectrometric data provided evidence for the existence of unique phosphorylated species containing between 1 and 7 phosphorylated serines. Our data do not preclude the presence of an 8th phosphorylated serine. In agreement with others (18, 25), we believe that the most abundant phosphorylation event is on S222. LeMay et al. also provided evidence for phosphorylation of S222 being present in the hyperphosphorylated NS5A form, as shown by a 36% reduction in hyperphosphorylation in the context of the S222A mutant. By generating an antiserum specific to phospho-S222, we here provide the first definitive evidence that phosphorylation of S222 is indeed a hallmark of hyperphosphorylated NS5A, since the antiserum showed very little reactivity to the basally phosphorylated NS5A species or the hyperphosphorylated S222A or S222D mutants (Fig. 8). However, despite the fact that S222 phosphorylation was detected predominantly in the hyperphosphorylated species, in our hands mutation at this residue did not affect the ratio of hyper- to basally phosphorylated NS5A (Fig. 7). This suggests that phosphorylation of S222 alone is neither the sole component of nor sufficient to produce the hyperphosphorylated species. Additionally, mutation of S222 to either A or D had no effect on RNA replication, the only phenotype being a modest yet significant reduction in the titer of released virus for S222D.

To complement the biochemical analysis of phosphorylation, we conducted an extensive mutagenesis of the identified phosphoacceptor residues. Many of these mutants had no phenotype, but this analysis pointed to a critical role for S229: both phosphoablatant and phosphomimetic mutations at this site abrogated replication of the subgenomic replicon, in agreement with the recent data of Fridell and coworkers (24). The observation that both types of mutations at this residue were lethal might suggest that although this serine residue is phosphorylated, it plays an additional role that is not dependent on phosphorylation. In contrast, and again in agreement with the work of Fridell (24), the S225A mutant had a partial defect in replication; this was restored by the S225D mutation, consistent with a role for phosphorylation at this residue.

None of the mutations within LCS I stimulated RNA replication, which is in contrast to the situation in the genotype 1b (Con1) subgenomic replicon (22). In fact, it appears that the genotype 2a mutations exhibit the phenotype opposite to that of the corresponding mutations in genotype 1b, despite the absolute conservation of the sequence (see Table S1 in the supplemental material). For example, mutations of S229 in genotype 1b significantly enhance replication, yet the same mutations are lethal in genotype 2a. These differences are hard to interpret but presumably are indicative of fundamental genotype-specific differences in the interactions between NS5A and other viral (or cellular) proteins during the process of virus genome replication.

### Evidence for sequential phosphorylation across LCS I.

During our analysis, we observed that for the phosphomimetic (S → D) mutations within LCS I, the mobility of the basally phosphorylated species appeared to vary between mutants, whereas the mobility of the hyperphosphorylated species did not change, with the exception of the S235D mutant, which exhibited a slight, but consistent increase in mobility. A clear trend emerged whereby, as the position of the phosphomimetic mutation was shifted further toward the C terminus of LCS I, there was a concomitant decrease in the mobility (increase in apparent molecular weight) of the basally phosphorylated species (Fig. 4). One interpretation of these data is that there is a sequential and ordered cascade of phosphorylation events across this region. In this scenario, a phosphorylated amino acid is an essential component of the recognition motif for a kinase that phosphorylates a proximal residue, either N- or C-terminal to the preexisting phosphorylation site. This phosphorylation event then serves to “prime” the phosphorylation of the next residue in a similar manner. In this scenario, phosphorylation at S238 would enable phosphorylation at S235 and so on. There are many examples of sequential phosphorylation cascades in cellular proteins: for example, GRASP65 (35) and PTEN (36). In the latter case, one cascade is not linear; for example, phosphorylation in PTEN proceeds from S385 → S380 → T383 → T382. Our data cannot rule out such a process in NS5A, and the identification of the phosphorylating kinases coupled with a sophisticated temporal NMR analysis would be required to address this question in more detail, as in the case of PTEN. Interestingly, another protein exhibiting sequential phosphorylation is β-catenin, which also interacts with NS5A (37). In β-catenin, S45 is phosphorylated by CKI, which primes it for subsequent phosphorylation at S41, S37, and S33 by GSK-3 (38). A similar scenario could be envisioned for NS5A, and intriguingly, S222 and S225 are predicted to constitute GSK3 phosphorylation sites. By mimicking phosphorylation, the S238D mutation would force sequential phosphorylation across the entire LCS I, whereas S → D mutations located N-terminal to S238 would only drive a subset of the possible phosphorylation events. This hypothesis is consistent with the notion that addition of multiple phosphates to LCS I would therefore contribute to the hyperphosphorylation phenotype. The fact that, in the case of wild-type NS5A, no intermediates between the basally and hyperphosphorylated forms are seen suggests that once the phosphorylation cascade begins, it proceeds to completion. Additionally, the presence of both forms implies that the phosphorylation cascade is an all-or-nothing series of events. NS5A species of intermediate apparent molecular weight can only form when the cascade is perturbed, for example, by introduction of a phosphomimetic mutant which overcomes any regulation of the phosphorylation cascade. However, even in the S238D mutant, where we see the largest increase in apparent molecular weight, the basally phosphorylated and hyperphosphorylated species can still be distinguished, so clearly additional phosphorylation events are necessary to generate the hyperphosphorylated species. One candidate is the other phosphoacceptor identified in this study, S146.

### S146 regulates hyperphosphorylation.

S146 is located within domain I and has not previously been identified as a phosphoacceptor. Mutation of this site had no effect on either genome replication or virus assembly; however, it was noteworthy that the S146D mutant exhibited a significant decrease in the abundance of hyperphosphorylated NS5A, decreasing from 42% (±0.6 standard error of the mean [SEM]) of total NS5A for the wild type to 17% (±1.0 SEM) for the S146D mutant. However, this was not accompanied by a concomitant increase in the abundance of the basally phosphorylated species, suggesting that in the absence of S222 phosphorylation, partially hyperphosphorylated NS5A might be subject to rapid degradation.

**FIG 9:**
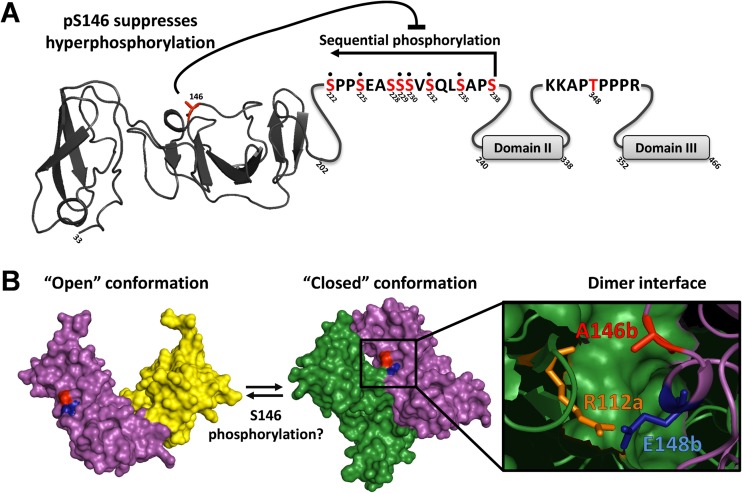
Summary and potential role of NS5A phosphorylation. (A) Schematic of NS5A showing the location and relationship of phosphorylation events identified. Domain I is denoted by the structure of the Con1 monomer (6); sequences of LCS I and LCS II are shown; domains II and III are denoted by gray boxes. Highlighted in red are those residues identified as phosphorylation sites. Note that residues S228 and S238 were not observed directly by mass spectrometry, but the presence of a heptaphosphorylated phosphopeptide with only 8 possible phosphorylation sites strongly indicates their presence. (B) The two differing dimeric conformations of NS5A domain I are shown, “closed” (3FQM) (5) and “open” (1ZH1) (6), green/purple and purple/yellow, respectively. The structural studies utilized a Con1 sequence where an alanine is present at position 146. Residue E148b (conserved in JFH-1 and Con1) forms an intramolecular bond with R112a on the opposite monomer, blue and orange, respectively (5). It is likely that phosphorylation at 146 would exert an effect on this key dimer interaction of the “closed” conformation.

The S146D mutant also exhibited a loss in reactivity to the phospho-S222 antiserum, consistent with the fact that S222 phosphorylation is a hallmark of the hyperphosphorylated species and is downregulated by the S146D mutation. Given that phosphomimetic mutations in LCS I appeared to at least partially drive the conversion of the basally phosphorylated species into the hyperphosphorylated species, we investigated whether these mutations could override the inhibitory effect of S146D on hyperphosphorylation. However, this was not the case, and furthermore, the addition of S146D was not able to block the increase in apparent molecular weight of the basally phosphorylated species that resulted from LCS I phosphomimetic mutations. We therefore propose that a number of factors contribute to the hyperphosphorylation of NS5A. First, sequential phosphorylation across LCS I is required, and second, hyperphosphorylation is negatively regulated by phosphorylation at S146 (Fig. 9A). We further propose, based on the observation that the S146D/S238D mutant still exhibits two species of distinct mobility, that additional (as yet undefined) phosphorylation events are required to produce the hyperphosphorylated species of NS5A.

Our data do not support the previously proposed hypothesis that hyperphosphorylation inhibits genome replication. We identify mutations that significantly reduce hyperphosphorylation yet have no effect on either genome replication or virus assembly and release (e.g., S146D and S225D). A caveat to this conclusion is that NS5A expression from replication-defective mutants (e.g., the S222/225A and S229A/D mutants) is below the Western blot detection threshold. Thus, it is formally possible that these mutants have high levels of hyperphosphorylation; however, at least for the S225A mutant, this appears not to be the case, since this mutant exhibits both low levels of replication and hyperphosphorylation. It may be that the high replicative capacity of JFH-1 overcomes any dependence on hyperphosphorylation which is seen in genotype 1b replicons (11, 22).

Interestingly, a serine at residue 146 is present only in genotype 1a and 2a isolates of HCV; in the majority of other isolates across all genotypes and subtypes (including 1b and 2b), this residue is an alanine, although the surrounding sequence is highly conserved. It is therefore possible that the presence of a phosphorylatable residue at this position might contribute to the enhanced replicative capacity of JFH-1 by allowing for an additional level of control of hyperphosphorylation. Further, albeit circumstantial, evidence supporting this hypothesis comes from an examination of the two published structures of domain I. As shown in Fig. 9B, there are two differing dimeric conformations of NS5A domain I, the “closed” conformation (3FQM) (5) and the “open” conformation (1ZH1) (6). Both structures utilized the genotype 1b Con1 sequence, in which an alanine is present at position 146. In the closed conformation, A146 is located close to the dimer interface, whereas in the open conformation, it is on the opposite face of each monomer. In the closed conformation, a proximal residue, E148 (conserved in both JFH-1 and Con1), forms an intramolecular contact via a hydrogen bond with R112 on the other monomer. While it is not possible to predict the effects of S146 phosphorylation on dimer formation, two scenarios are possible. First, the negative charge imparted by S146 phosphorylation could participate in an electrostatic interaction with R112 on the other monomer (Fig. 9B), potentially stabilizing the dimer interface. Alternatively, S146 phosphorylation could disrupt the interaction between R112 and E148, thereby promoting a switch from the closed to the open conformation. Whether phosphorylation of S146 might affect the NS5A conformation at the point of dimerization or be involved in switching between conformations of dimeric NS5A remains to be determined. To further support the potential involvement of S146 phosphorylation in regulating dimerization, we modeled the structural consequences of a glutamic acid substitution at position A146 using the Robetta full-chain protein structure prediction server. As shown in Fig. S7 in the supplemental material, this analysis predicted that phosphorylation at this residue could indeed project further into the dimer interface, consistent with a role for S146 phosphorylation in regulating dimerization; however, such analysis does not allow us to predict whether phosphorylation of S146 would favor the closed or open conformation.

In summary, our data provide novel insights into the complexity and regulation of NS5A phosphorylation. They provide a framework for future studies, which will seek to identify the kinases involved in these events and dissect the roles of the different phosphorylated forms of NS5A in the virus life cycle. Furthermore, NS5A phosphorylation is likely to be a dynamic process, therefore also requiring the action of protein phosphatases, such as PP2A, which has been shown to be upregulated by HCV infection (39) and to interact with NS5A (40). We hope that these studies will not only lead to a deeper understanding of NS5A function but provide opportunities for the development of new therapeutic options for HCV treatment.

## Supplementary Material

Supplemental material
