# Supplementary material for: Insights into the Complexity and Functionality of Hepatitis C Virus NS5A Phosphorylation
Source: J Virol. 2014 Feb;88(3):1421–32. doi: 10.1128/JVI.03017-13 (PMC3911623; doi:10.1128/JVI.03017-13)
Supplement: Supplemental material [file supp_88_3_1421__index.html]

Supplemental material 

# Insights into the Complexity and Functionality of Hepatitis C Virus NS5A Phosphorylation

## Supplemental material

**Files in this Data Supplement:**

- Supplemental file 1 -

  Fig. S1 to S4 (Identification of S146, S222, SS222/5, and T348 phosphorylation sites.)

  Fig. S5 (Effect of long-term passage on hyperphosphorylation of NS5A.)

  Fig. S6 (Phosphorylation of T348 does not affect the binding of SH3 domains to the P2 polyproline motif.)

  Fig. S7 (Structural prediction of a phosphomimetic at position 146 shows no significant alteration to NS5A domain I monomer structure.)

  Table S1 (Phenotype 57 of mutants in putative LCS I phosphorylation sites in the context of genotype 1b or 2a.)

  PDF, 973K
