# Supplementary material for: Insights into the Complexity and Functionality of Hepatitis C Virus NS5A Phosphorylation
Source: J Virol. 2014 Feb;88(3):1421–32. doi: 10.1128/JVI.03017-13 (PMC3911623; doi:10.1128/JVI.03017-13)
Supplement: Supplemental material [file JVI.03017-13_zjv999098599so1.pdf]

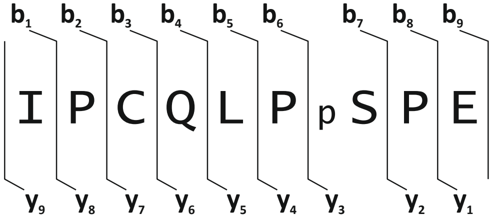

Seq.	b-ions		y-ions	
I	b1	**114.0913**	y9	-
P	b2	211.1441	y8	909.4135
C	b3	**371.1748**	y7	812.3607
Q	b4	**499.2333**	y6	652.3301
L	b5	**612.3174**	y5	524.2715
P	b6	709.3702	y4	**411.1874**
S	b7	**778.3916**	y3	**314.1347**
P	b8	875.4444	y2	**245.1132**
E	b9	-	y1	148.0604

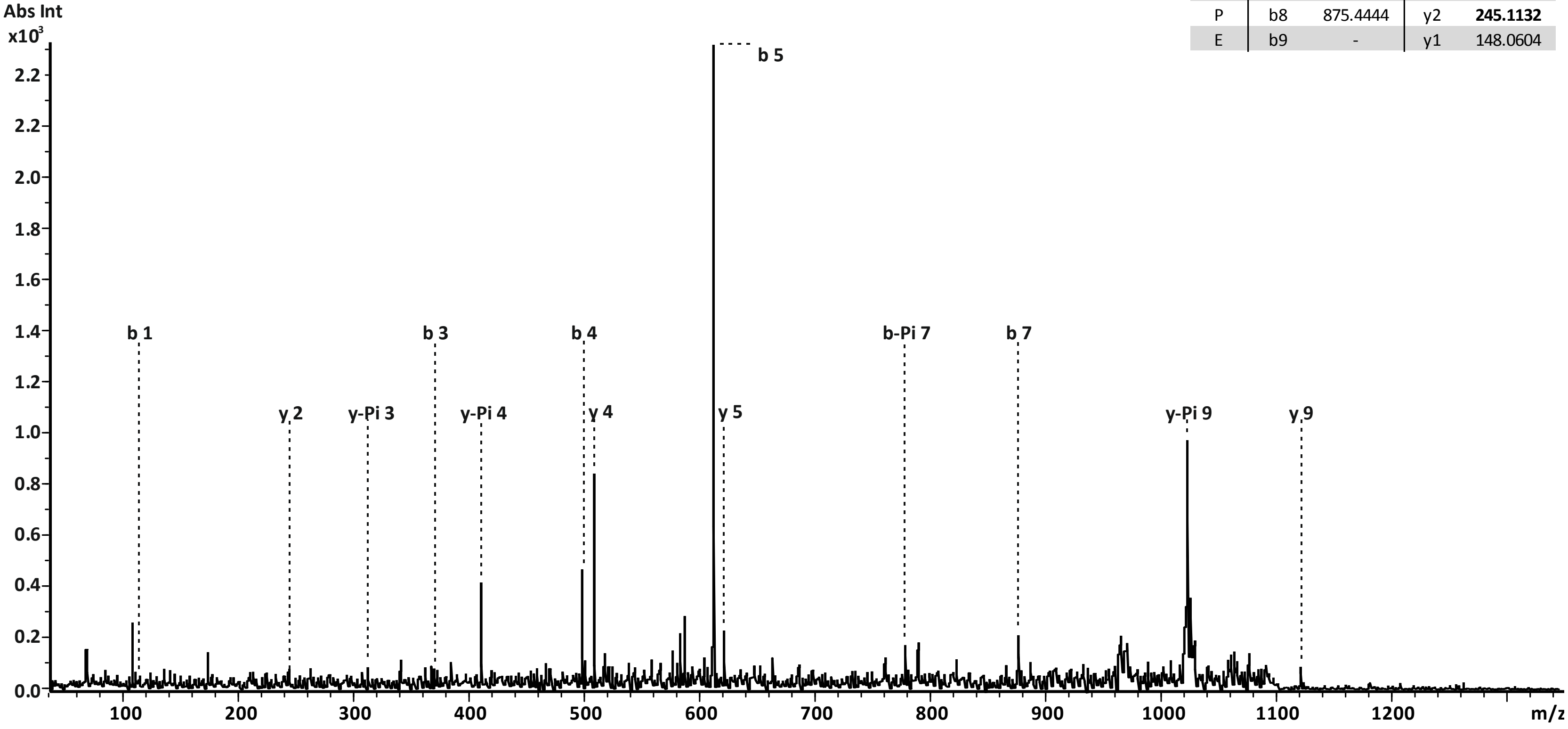

Figure S1

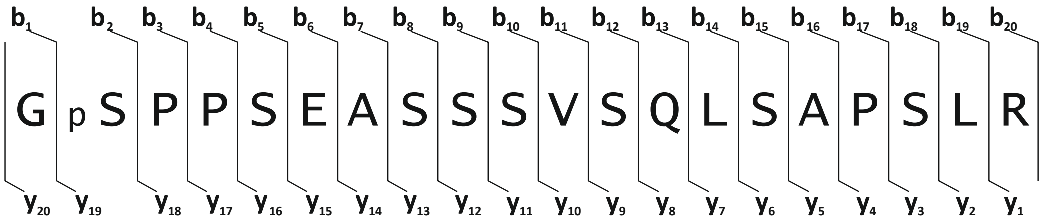

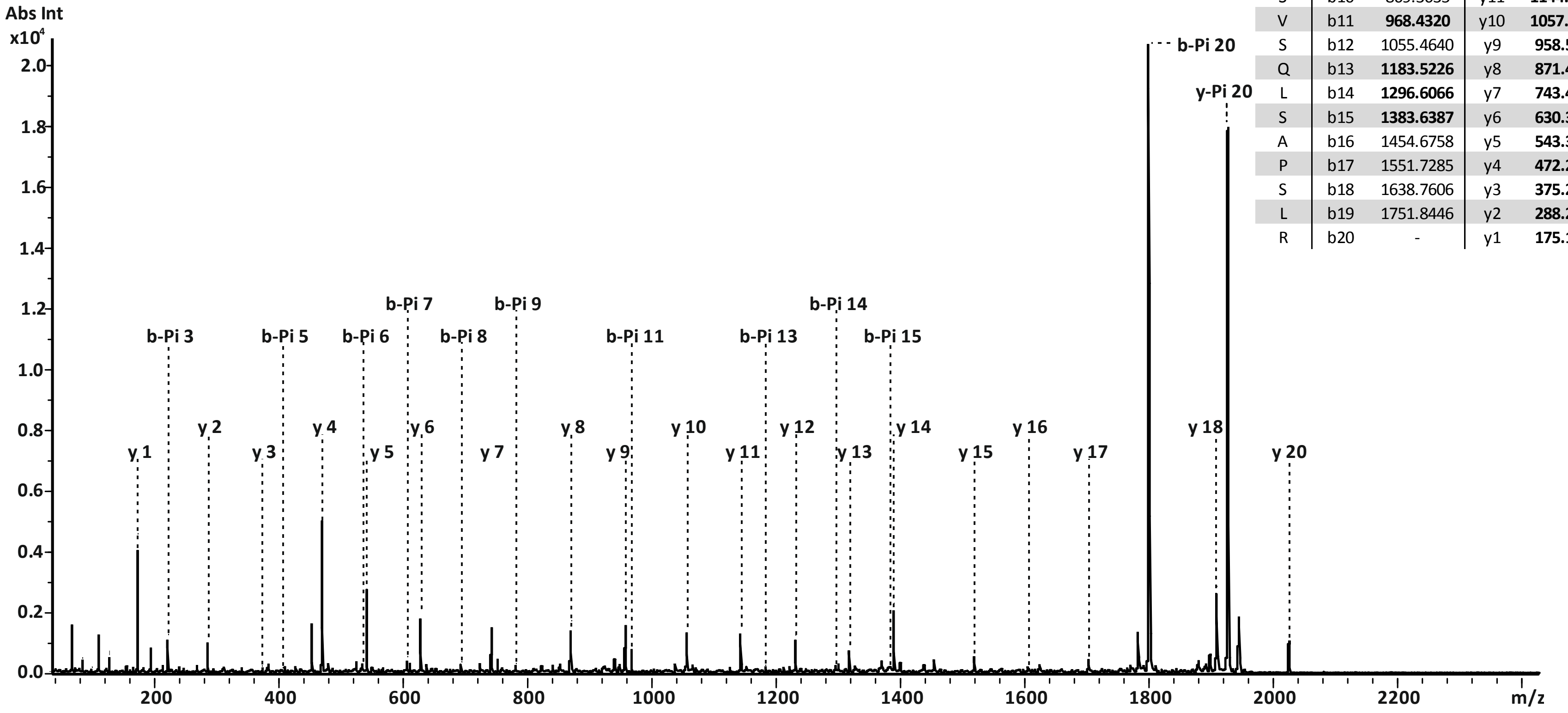

Seq.	b-ions		y-ions	
G	b1	58.0287	y20	-
S	b2	127.0502	y19	1868.9348
P	b3	224.1030	y18	1799.9134
P	b4	321.1557	y17	1702.8606
S	b5	408.1878	y16	1605.8079
E	b6	537.2304	y15	1518.7758
A	b7	608.2675	y14	1389.7332
S	b8	695.2995	y13	1318.6961
S	b9	782.3315	y12	1231.6641
S	b10	869.3635	y11	1144.6321
V	b11	968.4320	y10	1057.6000
S	b12	1055.4640	y9	958.5316
Q	b13	1183.5226	y8	871.4996
L	b14	1296.6066	y7	743.4410
S	b15	1383.6387	y6	630.3570
A	b16	1454.6758	y5	543.3249
P	b17	1551.7285	y4	472.2878
S	b18	1638.7606	y3	375.2350
L	b19	1751.8446	y2	288.2030
R	b20	-	y1	175.1190

Figure S2

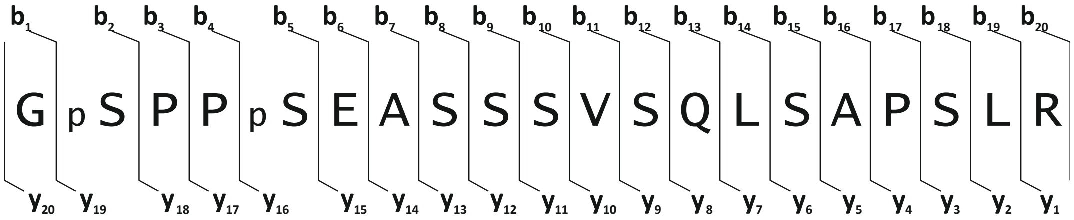

Seq.	b-ions		y-ions	
G	b1	58.0287	y20	-
S	b2	127.0502	y19	1850.9243
P	b3	**224.1030**	y18	**1781.9028**
P	b4	321.1557	y17	1684.8501
S	b5	390.1772	y16	1587.7973
E	b6	519.2198	y15	1518.7758
A	b7	590.2569	y14	**1389.7332**
S	b8	677.2889	y13	1318.6961
S	b9	764.3210	y12	**1231.6641**
S	b10	851.3530	y11	**1144.6321**
V	b11	**950.4214**	y10	**1057.6000**
S	b12	1037.4534	y9	**958.5316**
Q	b13	1165.5120	y8	**871.4996**
L	b14	1278.5961	y7	**743.4410**
S	b15	1365.6281	y6	**630.3570**
A	b16	1436.6652	y5	**543.3249**
P	b17	1533.7180	y4	**472.2878**
S	b18	1620.7500	y3	375.2350
L	b19	1733.8341	y2	**288.2030**
R	b20	-	y1	**175.1190**

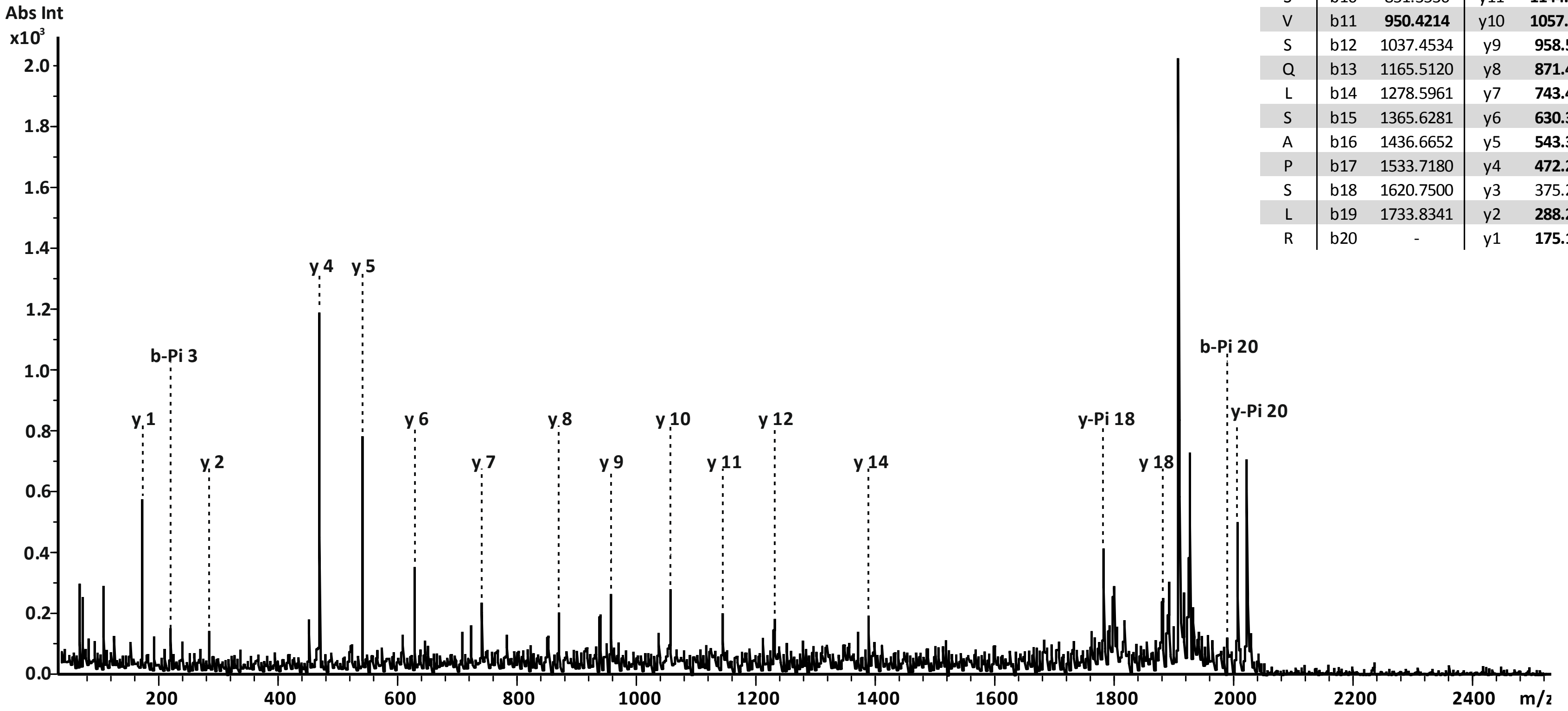

Figure S3

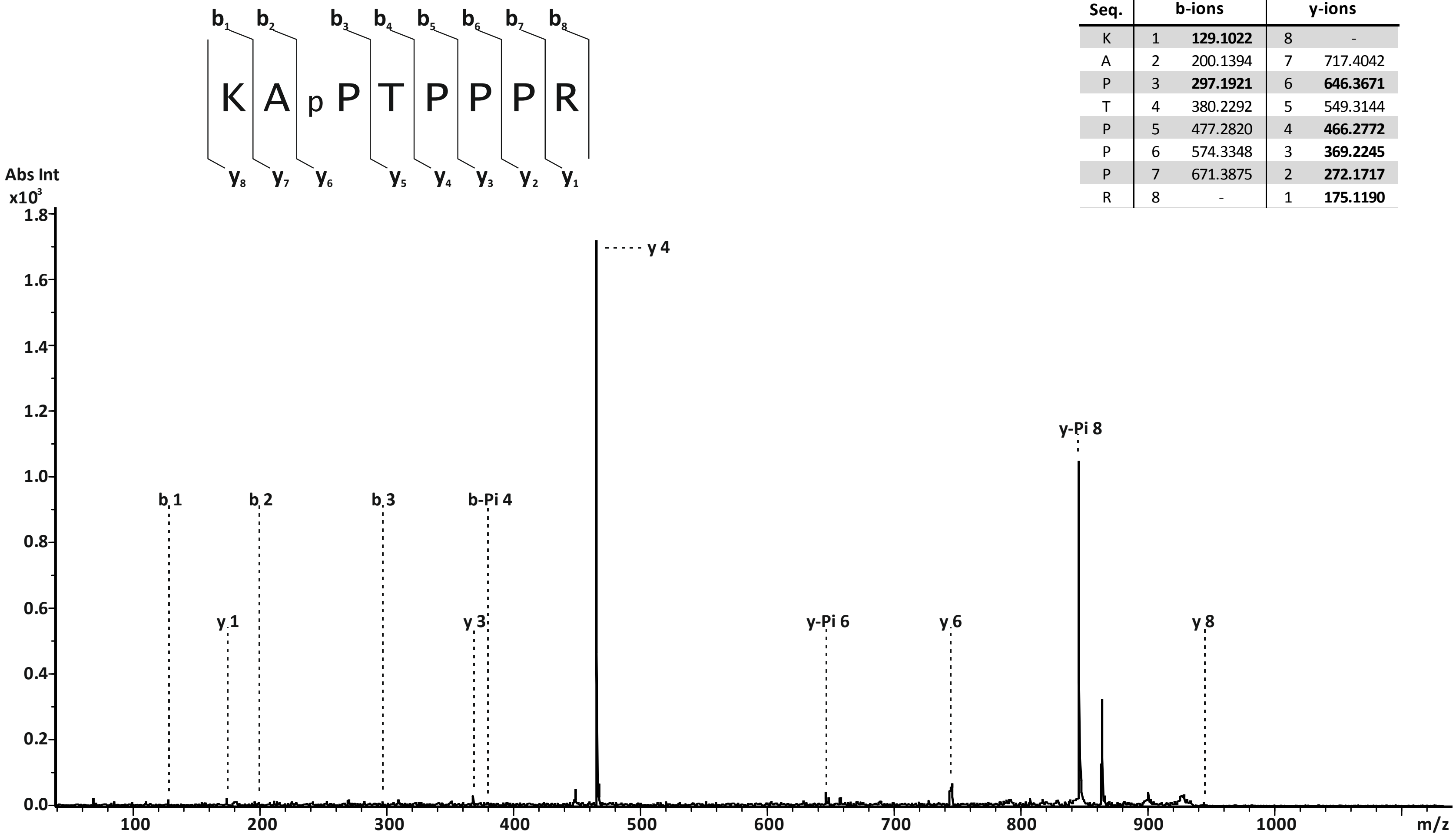

Seq.	b-ions		y-ions	
K	1	**129.1022**	8	-
A	2	200.1394	7	717.4042
P	3	**297.1921**	6	**646.3671**
T	4	380.2292	5	549.3144
P	5	477.2820	4	**466.2772**
P	6	574.3348	3	**369.2245**
P	7	671.3875	2	**272.1717**
R	8	-	1	**175.1190**

Figure S4

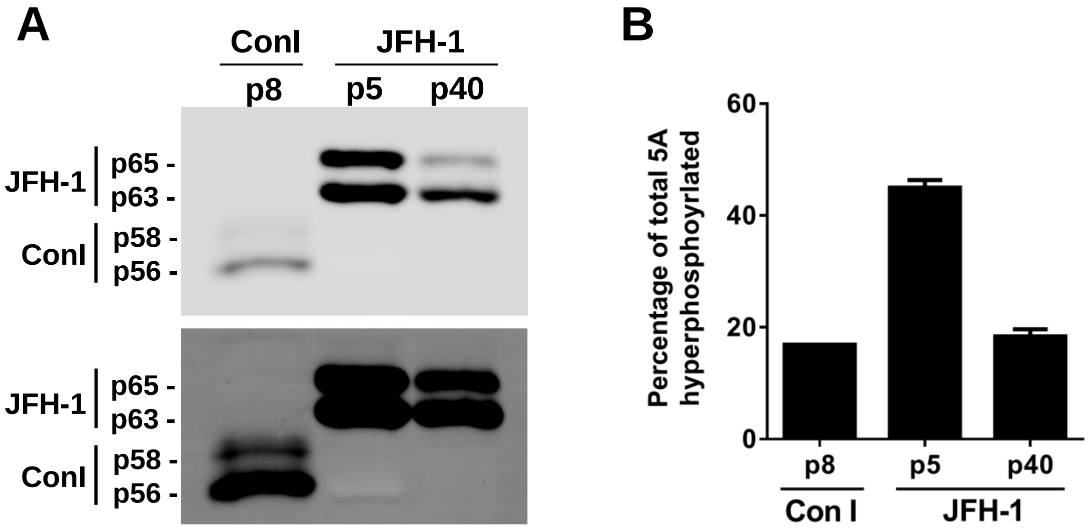
A
ConI
JFH-1
p8
p5
p40
JFH-1
p65 -
p63 -
ConI
p58 -
p56 -
JFH-1
p65 -
p63 -
ConI
p58 -
p56 -
B
Percentage of total 5A hyperphosphoyrlated
60
40
20
0
p8
p5
p40
Con I
JFH-1

Figure S5

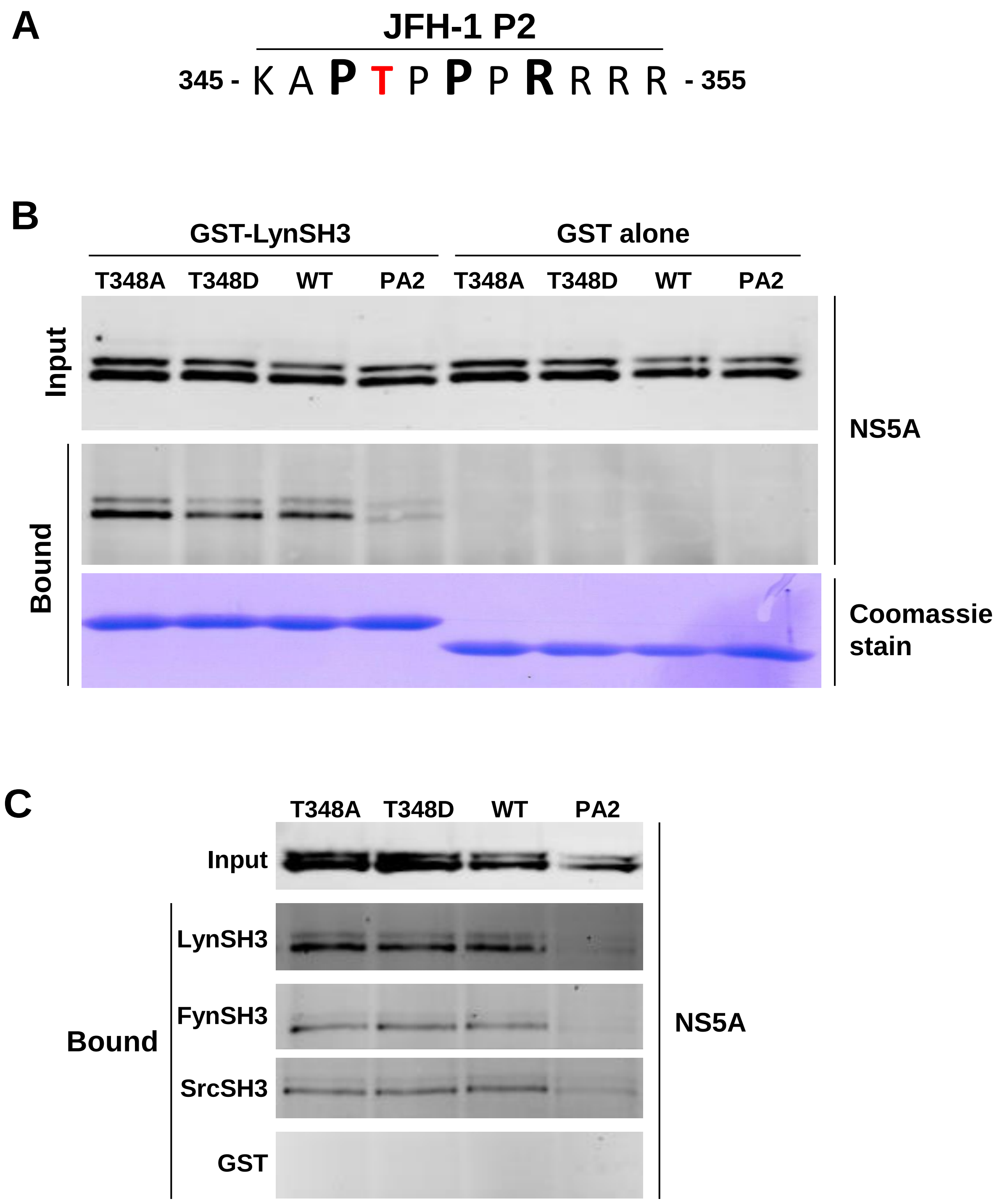
A
JFH-1 P2
345 - K A P T P P P R R R R - 355
B
GST-LynSH3
GST alone
T348A T348D WT PA2 T348A T348D WT PA2
Input
Bound
NS5A
Coomassie stain
C
T348A T348D WT PA2
Input
LynSH3
FynSH3
SrcSH3
GST
Bound
NS5A

Figure S6

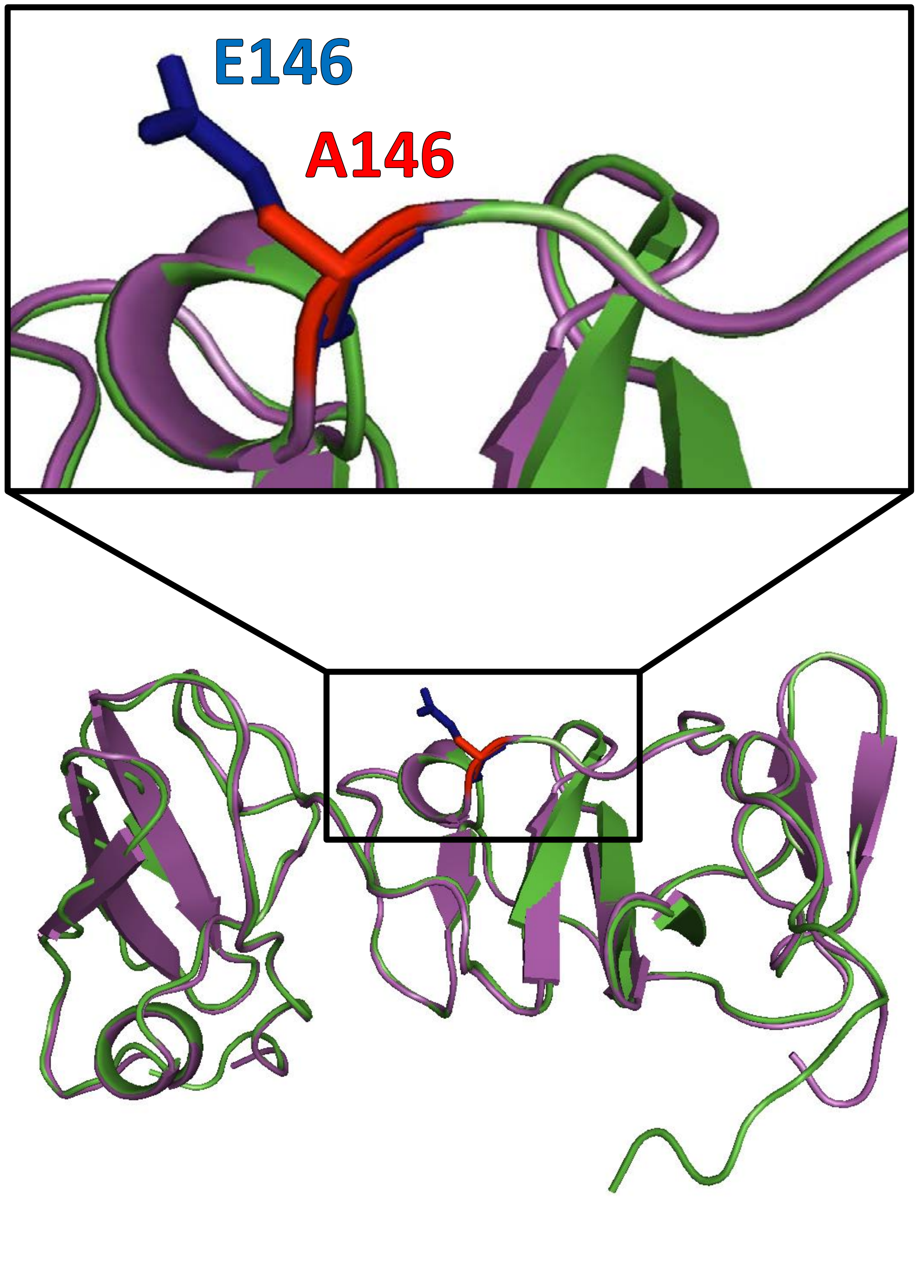

Figure S7

Supplementary Figure legends.

Figure S1. Identification of S146 phosphorylation site. MS/MS spectra of precursor ion 1120 m/z, analysed by Mascot software (Matrix Science). B and y ions observed are shown on the MS/MS spectra as well as highlighted in the table as bold. Illustration of the phosphopeptide 1120 with the location of phosphorylation site indicated.

Figure S2. Identification of S222 phosphorylation site. MS/MS spectra of precursor ion 2023 m/z, analysed by Mascot software (Matrix Science). B and y ions observed are shown on the MS/MS spectra as well as highlighted in the table as bold. Illustration of the phosphopeptide 2023 with the location of phosphorylation site indicated.

Figure S3. Identification of SS222/5 phosphorylation sites. MS/MS spectra of precursor ion 2103 m/z, analysed by Mascot software (Matrix Science). B and y ions observed are shown on the MS/MS spectra as well as highlighted in the table as bold. Illustration of the phosphopeptide 2103 with the location of phosphorylation site indicated.

Figure S4. Identification of T348 phosphorylation site. MS/MS spectra of precursor ion 943 m/z, analysed by Mascot software (Matrix Science). B and y ions observed are shown on the MS/MS spectra as well as highlighted in the table as bold. Illustration of the phosphopeptide 943 with the location of phosphorylation site indicated.

Figure S5. The effect of long term passage on the hyperphosphorylation of NS5A.

SGR-neo-JFH-1 or Con1 RNAs were electroporated into Huh7 cells and stable cell lines subjected to G418 selection (1 mg/ml) for 20 days, followed by a 1:5 passage every 3 days at 0.5 mg/ml G418. **(A)** After the indicated number of passages cells were lysed in GLB and lysates analysed by SDS-PAGE/Western blot probing for NS5A (9E10). The difference in the apparent molecular weight of Con1 and JFH-1 results from the 18 a.a. insertion in domain III of NS5A. **(B)** The percentage of total NS5A in the hyperphosphorylated species of ConI and JFH-1 NS5A, p58 and p63 respectively, was quantified. After long term passage there was a drop in the amount of NS5A phosphorylated from 45% to 18%. n=2, ** p <0.01 significance

Figure S6. The phosphorylation of T348 does not affect the binding of SH3 domains to the P2 polyproline motif. **(A)** Sequence of the phosphopeptide from LCS II showing the conserved prolines (bold) and the phosphorylated threonine residue (red). **(B)** Lysates from Huh7 cells electroporated with the indicated subgenomic replicons were subjected to GST pulldown using either GST-LynSH3 (lanes 1-4) or GST alone (lanes 5-8). 5% of the input lysate and the total bound fraction were analysed by western blot with an anti-NS5A antiserum (sheep). Equivalent loading of the GST fusions was verified by Coomassie staining (lower panel). PA2 refers to a mutation of the two prolines highlighted in (A) to alanine [1]. **(C)** As **(B)** but lysates were subjected to GST pulldown using either GST-LynSH3, FynSH3 or SrcSH3.

Figure S7. Structural prediction of a phosphomimetic at position 146 shows no significant alteration to NS5A domain I monomer structure. The NS5A ConI sequence used in both the Tellinghuisen [2] and Love [3] studies was computationally modelled with a glutamic acid substitution at position A146 using the Robetta full-chain protein structure prediction server. Five full structure predictions

were calculated and model 1 (green) which showed the highest similarity to existing monomer structures was aligned with the Tellinghuisen et al monomer (magenta) in PyMol. Residues A146 (red) and E146 (blue) are highlighted. No dramatic shift in model 1 structure was observed as a result of the A146E substitution, reflecting the observation that position 146 resides on an external, non-helical/non-beta-sheet stretch of the peptide chain.

Supplementary Table S1 Phenotype of mutants in putative LCS I phosphorylation sites in the context of genotype 1b or 2a.

Residue number:				
**NS5A**	**Polyprotein***	**Gt 2a (this study)**	**Gt 2a [4]**	**Gt 1b [5]**
S222	2194	A wildtype	A wildtype	A wildtype
		D wildtype	D not tested	E wildtype
S225	2197	A down 6 fold	A down 3 fold	A Up 6 fold
		D wildtype	E wildtype	E wildtype
S228	2200	A wildtype	A wildtype	A wildtype
		D wildtype	D not tested	E wildtype
S229	2201	A down >1000 fold	A no replication	A up >10 fold
		D down >1000 fold	E no replication	E up 50 fold
S230	2202	A wildtype	A wildtype	A up 5 fold
		D wildtype	D not tested	E wildtype
S232	2204	A down 10 fold	A down 10 fold	A up >10 fold
		D wildtype	E wildtype	E wildtype
S235	2207	A not tested	A no replication	A up >10 fold
		D wildtype	E wildtype	E wildtype
S238	2210	A not tested	A wildtype	A wildtype
		D wildtype	D not tested	E wildtype

* Gt 1b polyprotein numbering

Supplementary References

1. Hughes M, Gretton S, Shelton H, Brown DD, McCormick CJ, Angus AG, Patel AH, Griffin S, Harris M (2009) A conserved proline between domains II and III of hepatitis C virus NS5A influences both RNA replication and virus assembly. J Virol 83: 10788-10796.

2. Tellinghuisen TL, Marcotrigiano J, Rice CM (2005) Structure of the zinc-binding domain of an essential component of the hepatitis C virus replicase. Nature 435: 374-379.

3. Love RA, Brodsky O, Hickey MJ, Wells PA, Cronin CN (2009) Crystal structure of a novel dimeric form of NS5A domain I protein from hepatitis C virus. J Virol 83: 4395-4403.

4. Fridell RA, Valera L, Qiu D, Kirk MJ, Wang C, Gao M (2013) Intragenic complementation of hepatitis C virus NS5A RNA replication-defective alleles. J Virol 87: 2320-2329.

5. Appel N, Pietschmann T, Bartenschlager R (2005) Mutational analysis of hepatitis C virus nonstructural protein 5A: potential role of differential phosphorylation in RNA replication and identification of a genetically flexible domain. J Virol 79: 3187-3194.
